# Supplementary figures and images for: Exploring patient perspectives: A qualitative inquiry into healthcare perceptions, experiences and satisfaction in Lebanon
Source: PLoS One. 2023 Aug 17;18(8):e0280665. doi: 10.1371/journal.pone.0280665 (PMC10434906; doi:10.1371/journal.pone.0280665)

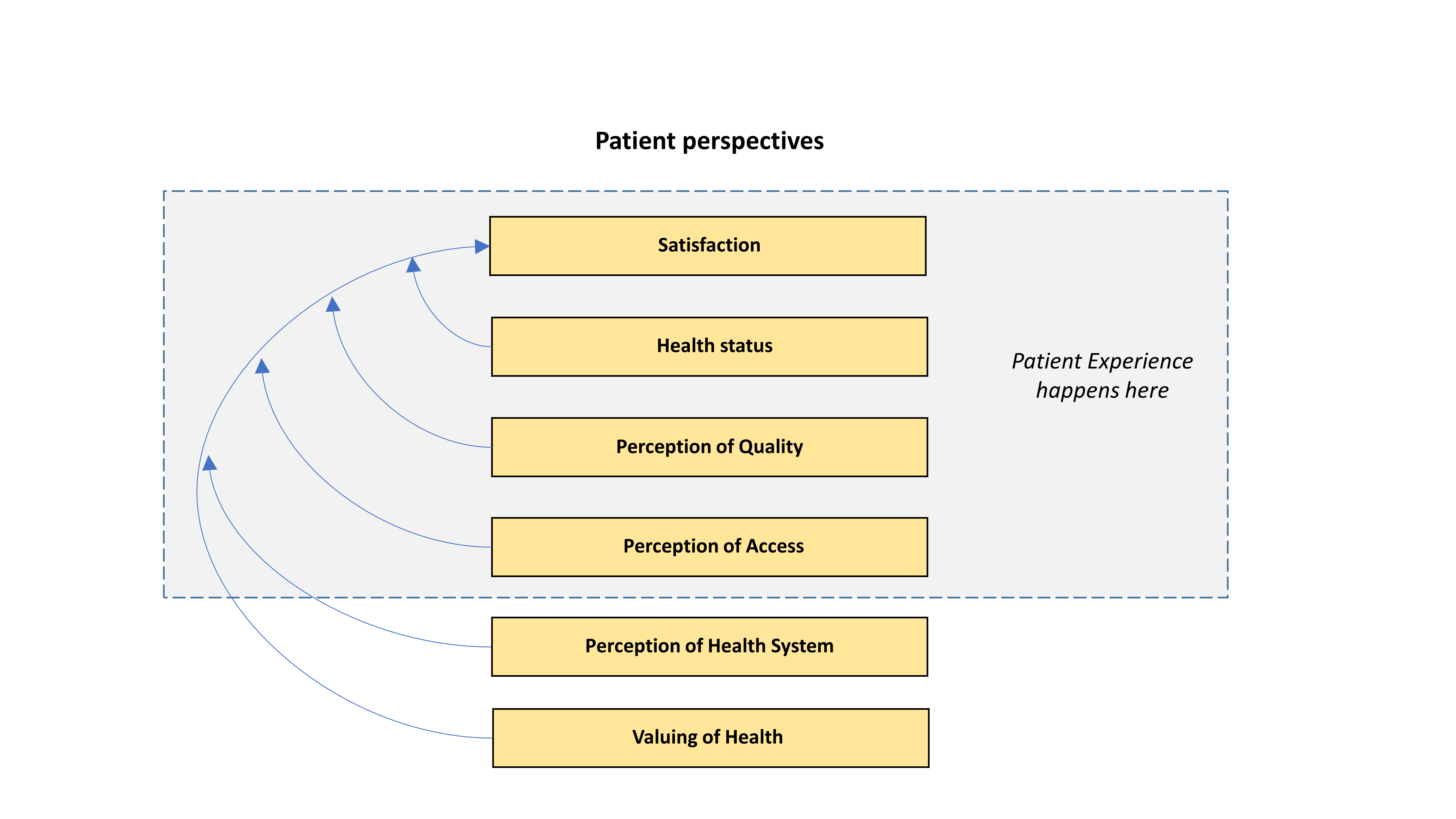

Supplement: S1 Fig — (TIF) [file pone.0280665.s001.tif]
