# Supplementary material for: Exploring patient perspectives: A qualitative inquiry into healthcare perceptions, experiences and satisfaction in Lebanon
Source: PLoS One. 2023 Aug 17;18(8):e0280665. doi: 10.1371/journal.pone.0280665 (PMC10434906; doi:10.1371/journal.pone.0280665)
Supplement: S1 Appendix — (DOCX) [file pone.0280665.s002.docx]

# **Appendix**

The key questions included in the discussion guide are listed below.

Q1. What does health

mean to you?

Q2. Please describe the health care offered in this area? Probe on hospitals and primary care

Q3. What characterizes the services that you have experienced so far? Probe on hospitals and primary care

Q4. How would you describe a ‘good hospital stay’? Probe on the role of different components (treatment, personal interaction, hygiene etc.)

Q5. How would you describe a ‘bad stay in hospital’? Probe the role of different components (treatment, personal interaction, hygiene etc.)

Q6. What information about a hospital stay do people need when they are admitted?

Q7. What would make a person visit the same hospital again?
